# Hospitalized children with influenza virus: a 17 year-long observational study including the 2009 H1N1 influenza pandemic and COVID-19 pandemic

**DOI:** 10.1186/s12879-026-12818-5

**Published:** 2026-02-12

**Authors:** Hedda Trømborg Jalving, Andreas Christensen, Svein Arne Nordbø, Kari Risnes, Henrik Døllner, Inger Heimdal

**Affiliations:** 1 Department of Clinical and Molecular Medicine, Norwegian University of Science and Technology (NTNU), Trondheim, Norway; 2 Children’s Clinic, St. Olavs Hospital, Trondheim University Hospital, Trondheim, Norway; 3 Department of Medical Microbiology, St. Olavs Hospital, Trondheim University Hospital, Trondheim, Norway

**Keywords:** Influenza, Children, Influenza A(H1N1)pdm09 pandemic (swine flu), COVID-19, Disease severity

## Abstract

**Background:**

Influenza viruses (IFV) have major impacts on children’s health. We studied IFV A/B epidemiology and disease severity during a 17 year-long period including the 2009 H1N1 influenza pandemic and post-COVID-19 in 2021–2023.

**Methods:**

Nasopharyngeal samples from children referred with fever or respiratory symptoms to a Norwegian hospital from 2006 to 2023 were tested with PCR for IFV A/B and 17 other pathogens. We compared IFV hospitalization rates and disease severity before and during the 2009 influenza pandemic, and before and after the COVID-19 pandemic, respectively, using age-adjusted logistic regression analyses.

**Results:**

From 2006 to 2023, 502 children with IFV A/B were included (IFV A 74%, IFV B 26%). Two-hundred-fifty-one (50%) were hospitalized > 24 h. During the 2009 H1N1 influenza pandemic rates of hospitalization > 24 h in children ≤ 5 and > 5 years of age increased from 44 to 72, and 4 to 20 per 100,000, respectively, compared to 2006–2009. The median age of IFV positive children doubled from 23 to 49 months (*p*=.006), but no other clinical changes occurred. Post-COVID-19 (2021–2023), the hospitalization rates only increased in children > 5 years from 12 to 25 per 100,000, and the median age almost doubled from 33 to 62 months (*p*<.001) compared to 2010–2020. Post-COVID-19, fewer children had lower respiratory tract infections (aOR 0.32, 95% CI 0.20–0.53) and severe complications (aOR 0.24, 95% CI 0.07–0.82).

**Conclusions:**

Compared to other influenza seasons, disease severity during the 2009 H1N1 influenza pandemic was unchanged, but post-COVID-19 children referred to hospital with IFV A/B had milder disease.

**Supplementary Information:**

The online version contains supplementary material available at 10.1186/s12879-026-12818-5.

## Background

Influenza virus (IFV) is an important contributor to pediatric morbidity and mortality [1]. Most infected children present with self-limiting respiratory symptoms, fever and impaired general condition [2, 3]. However, a few may experience complications necessitating hospital evaluation due to lower respiratory tract infections (LRTI), bacterial superinfections, and various CNS-complications, including febrile seizures and encephalitis [4]. The yearly (seasonal) epidemiology of IFV type A and B is driven by minor changes in the virus genome known as antigenic drift. A major change in the IFV A genome, known as antigenic shift, might lead to pandemics such as the 2009 H1N1 influenza pandemic. Children were especially susceptible to the A(H1N1)pdm09 virus due to immature immunity, although studies suggest that IFV was not associated with more severe disease in children during the 2009 H1N1 influenza pandemic than seasonal influenza [5]. In addition to genetic changes, the epidemiology of influenza viruses is influenced by various factors. For instance, the emergence of SARS-CoV-2 in late 2019, which led to the COVID-19 pandemic, reshaped the landscape of many pediatric infections, including influenza [6]. First, studies reported a decline in influenza cases in 2020 and 2021 [7–9] and subsequently, after lifting non-pharmacological interventions (NPIs) in 2021–2022, the number of influenza sick children surged again in Norway [10] and other countries [11–14]. These changes were likely to be caused by absent exposure to seasonal IFV and subsequently reduction in protective immune responses in the child population compared to pre-pandemic times, as it recently has been shown for IFV A in Norwegian children [15], but increased testing and possibly changes in healthcare-seeking behaviors might also contribute. Studies providing clinical data during these substantial alterations in epidemiology and immunology are sparse, although some reports suggest a higher age distribution and increased disease severity of IFV in children after COVID-19 [16].

We aim to evaluate how the 2009 H1N1 influenza pandemic and COVID-19 pandemic influenced the pediatric epidemiology and clinical course of IFV A/B, compared to regular influenza seasons. Using an extensive 17‑year dataset (2006–2023) of pediatric hospital admissions at the Children’s Clinic, St. Olavs Hospital, Norway, this study provides a unique possibility to compare seasonal trends, hospitalization rates, age distributions and measures of disease severity across both the 2009 H1N1 influenza pandemic and the COVID‑19 pandemic.

## Methods

### Study setting and population

This study is expansion of a larger single-center prospective observational study conducted at the Children’s Clinic, St. Olavs Hospital, Trondheim University Hospital in Norway from November 2006 to September 2017. St. Olavs Hospital is the sole hospital for all children (*n* = 60,108) living in the entire Sør-Trøndelag county in Mid-Norway. In short, immunocompetent children < 16 years of age presenting with respiratory symptoms were invited to participate if a nasopharyngeal sample was collected at the Children’s Clinic for clinical purposes. All children were referred after initial evaluation by a physician in primary health care. Additionally, IFV positive nasopharyngeal samples obtained from referred immunocompetent children during November 2026 to September 2023 with influenza-like symptoms were retrospectively considered for inclusion (Supplementary Fig. 1).

### Clinical evaluation and disease classification

Included children were routinely diagnosed and treated by physicians at Children’s Clinic either as out-patients, defined as a contact of less than 24 h, or (hospitalized) in-patients. We used standardized forms to collect clinical information from caregivers and physicians. Study members supplemented missing clinical information retrospectively from electronic medical records. Children were classified with either a sole upper respiratory tract infection (URTI), or a LRTI with or without URTI. Chronic disease includes asthma, other lung diseases, cerebral palsy, epilepsy, or heart condition. Prematurity was defined as gestational age < 36 weeks.

Clinical complications were retrospectively categorized as either mild or severe according to the authors’ evaluations. Mild complications included conditions such as simple febrile seizures and myositis. Any child admitted to the Pediatric Intensive Care Unit (PICU) was classified as having a severe complication. Additionally, severe complications encompassed complex febrile seizures, sepsis-like illnesses, osteomyelitis, and other serious conditions. The severity of the RTI episodes in hospitalized children was assessed with a non-validated severity score including (1) need of respiratory support (1–6 points), (2) use of intravenous fluids or a nasogastric feeding tube (2 points), and (3) length of stay ≥ 5 days (2 points). For children requiring respiratory support, we differentiated between oxygen therapy to maintain oxygen saturation ≥ 93% (1 point), high-flow nasal cannula (2 points), continuous positive airway pressure and bilevel positive airway pressure (3 points), noninvasive positive pressure ventilation with synchronized assisted ventilator pressure and pressure control as standard setting (4 points) and invasive respirator (6 points). Only the highest treatment modality was calculated if a child received several respiratory support modalities. A score ≥ 3 corresponding to or above the 75th percentile among all virus-positive children with RTI was defined as a “severe disease.”

### Laboratory methods

Nasopharyngeal aspirates collected from 2006 to 2017 were analyzed for 17 viruses with semiquantitative in-house real-time PCR and cultivated in conventional cell lines for the detection of respiratory viruses including IFV A and B as previously described [17]. Additionally, the growth of *Hemophilus influenzae*, *Moraxella catarrhalis* and *Streptococcus pneumoniae* in standard agarose media was recorded. From 2017, an increasing percentage of nasopharyngeal samples were collected as swabs, and after February 2020 only swaps were used. Samples collected from 2017 to 2023 were mainly analyzed with the same in-house PCR methods, which from 2020 also included SARS-CoV-2. From September 2019, samples received after closing hours at the microbiological laboratory were analyzed with QIAstat-DX Respiratory or QIAstat-DX Respiratory SARS-CoV-2 panel. Additionally, three rapid panels, including IFV A and B, became available in 2022: Alinity m Resp 4-plex from Abbot, Cobas^®^Liat from Roche and Xpert^®^ Xpress SARS-CoV-2/Flu/RSV from Cepheid. For all PCR analyses, a Ct value > 40 was regarded as negative.

### Statistical analysis

Age differences were tested by use of Mann-Whitney U and Chi-squared tests. We compared occurrence, clinical characteristics, clinical outcomes and microbiological variables of children diagnosed with influenza virus before (2006–2008) and during the 2009 H1N1 influenza pandemic and before and after the COVID-19 pandemic, (2010–2020 and 2021–2023) respectively, with multivariable logistic regression analyses, adjusting for age or detection of IFV A/B.

An epidemiological year was defined consistently from 1st August to 31th July the following year, except for 2006/2007 which began 1st November. Influenza detections were compared to the total number of nasopharyngeal samples performed at the Children’s Clinic. Hospitalizations rates were calculated as number of confirmed influenza cases in children hospitalized > 24 h per 100,000 children in Sør-Trøndelag county from epidemiological years 2006/2007 to 2022/2023. Population data was retrieved from Statistics Norway.

Statistical analyses were conducted in IBM SPSS Statistics version 29.0.1 and R. Figures and illustrations were made in R and Adobe Illustrator.

### Ethics approval and consent to participate

This study is approved by the Regional committee for medical and health research ethics (REC) Central Norway (No.29331). Children were enrolled in this study either prospectively or retrospectively. For prospective inclusion, parents/legal guardians – and the child when age appropriate i.e. 12 years or older – received both oral and written information about the study. Written informed consent was obtained from the parents/legal guardians of the participants during the hospital stay. Due to practical constraints, not all eligible children were invited during their hospitalization. These children were invited to participate retrospectively. An information letter about the study was sent by mail to the parents/legal guardians, and children aged 12 or older received an age-appropriate version. REC granted permission for retrospective inclusion using passive consent, meaning participation was assumed unless refusal was communicated within two weeks (2006–2017) or four weeks (2017–2023) after receiving the letter. All participants, whether included prospectively or retrospectively, retained the right to withdraw from the study at any time.

## Results

### Patient characteristics

IFV A and IFV B were identified in 3.9% (502/12,667) of nasopharyngeal samples collected from children referred with respiratory symptoms or other influenza suspect symptoms at Children’s Clinic from 2006 to 2023. Five children were included with more than one episode. Yearly number of total detections varied from 0 (2020/2021) to 87 (2022/2023) with a yearly average of 31 (95% CI 23–39). IFV A constituted 74% (370/502) and IFV B 26% (133/502), including one sample positive for both. Nearly eight out of ten had a high viral genomic load (Ct < 28), three out of four were single detections, and three out of four had positive bacterial cultures (Table 1).

**Table 1:** Clinical and microbiological data of influenza-positive children (2006–2023)

	Influenza virus A and B		Type A		Type B	
	(*N* = 502)		(*n* = 369)		(*n* = 132)	
	*n*	%	*n*	%	*n*	%
**Background characteristics**						
Median age in months (IQR)	36	(17–91)	31*	(15–73)*	79*	(26–115)*
Age < 2 years old	184	37	156	42	29	22
Female	213	42	157	43	56	42
Chronic disease^1^	102	20	72	20	29	22
Premature birth^2^	56/364	15	40/268	15	15/95	16
**Clinical data**						
Respiratory tract infection	489	97	358	97	130	98
Sole upper respiratory tract infection	246	50	170	48	76	58
Lower respiratory tract infection	243	50	188	52	54	42
Hospitalized > 24 h	251	50	191	52	59	45
Severe disease^3^	75	15	60	16	15	11
Length of stay ≥ 5 days	66	13	50	14	16	12
Admitted to intensive care unit	39/498	8	33/365	9	6	5
Fever (temperature > 38.5 °C)	212/493	43	164/364	44	47/128	37
Antibiotics	133	27	108	29	25	19
Complications	143/498	29	98/365	27	45	34
Severe complications	38/498	8	33/365	9	5	4
Invasive group A streptococcal disease	2/498	0.4	2	1	0	0
**Microbiological data**						
Influenza virus Ct value < 28^4^	372/478	78	274	78	97/125	78
Single influenza virus detection	375	75	268	73	107	81
Co-detection with other viruses	127	25	101	27	25	19
Bacterial co-detection^5^	163/212	77	124/157	79	39/55	71

Abbreviations: IQR, interquartile range; Ct, cycle threshold

* *p *< 0.001 by Mann-Whitney *U* test

^1^Chronic disease includes asthma or other lung disease, cerebral palsy, epilepsy or heart condition

^2^Gestational age < 36 weeks

^3^As defined by a severity score including hospitalization length > 5 days, need for respiratory support and treatment with supplemental fluids. A “severe disease” was defined as a score ≥ 75-percentile among all virus-positive children

^4^Ct values were mainly obtained from in-house real-time PCR analysis and QIAstat-DX Respiratory panels

^5^Hemophilus influenzae, Moraxella catarrhalis or Streptococcus pneumoniae

More males than females were referred and approximately one out of five had a chronic disease and one out of seven were born prematurely. Half of the children referred were treated as out-patients, half were hospitalized > 24 h, and one out of eight had a hospital stay ≥ 5 days. Half were diagnosed with LRTI and half with a URTI only, and one fourth received antibiotics. One out of four developed complications, one out of seven had a severe disease as defined by a severity score ≥ 3, and one out of 12 were diagnosed with a severe complication (Table 1, supplementary Table 1).

Children with IFV A were younger than children with IFV B (median age: 31 vs. 79 months, Table 1). Children with IFV A were more often given antibiotics (OR 1.8, 95% CI 1.1-3.0, adjusted by age), but there were no other differences in demographic, clinical and microbiological variables between IFV A and IFV B (data not shown).

### Influenza seasonality

**Fig. 1:**
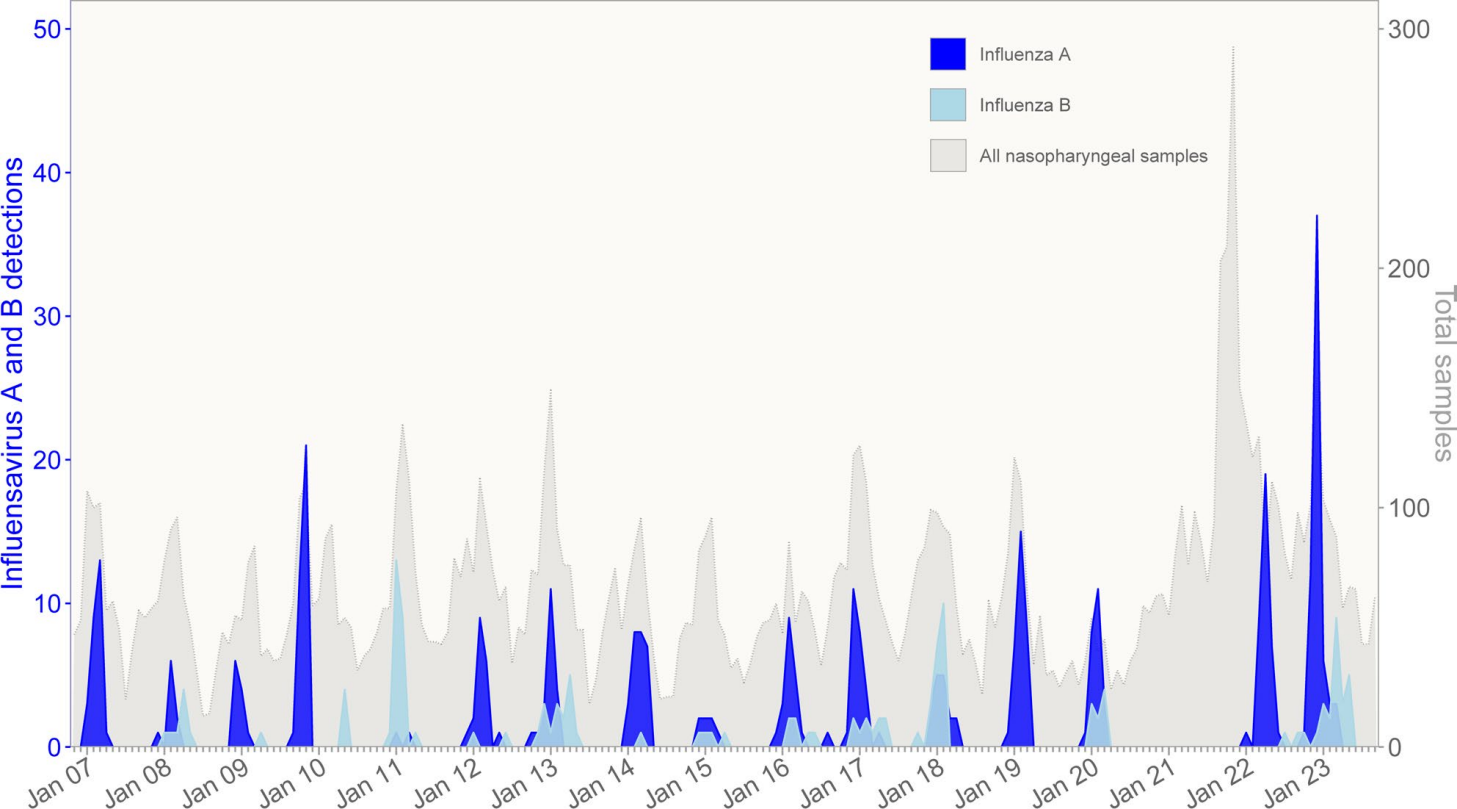
Number of influenza virus A (dark blue) and influenza virus B (light blue) positive children by month from November 2006 to September 2023 (left y axis). Number of upper respiratory tract samples tested for influenza virus at the Children’s Clinic, St. Olavs hospital during the same time period are shown in light gray, corresponding to the right y axis

**Fig. 2:**
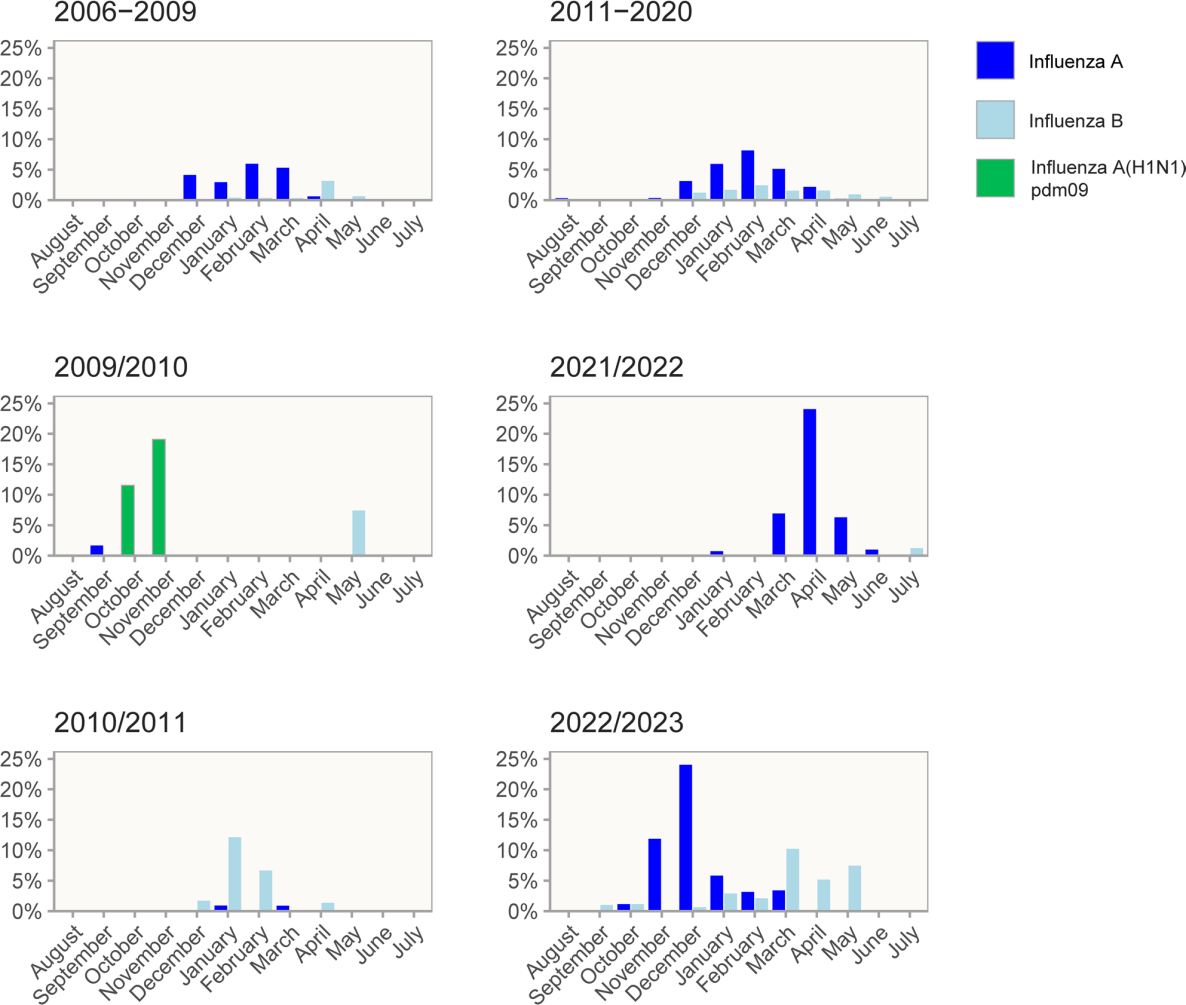
Seasonal variation of influenza virus A (dark blue), influenza virus B (light blue) and influenza virus A(H1N1)pdm09 (green). Hight of the bars corresponds to detection of influenza virus as a percentage of all upper respiratory tract samples taken at the Children’s Clinic, St. Olavs hospital

The detection patterns differed between IFV A and B. Before COVID-19, IFV A had a homogeneous detection pattern with yearly epidemics from November to March with an average of 12 hospitalizations each year (Figs. 1 and 2). Two seasons differed, however: in 2009/2010, there was a relatively early (October and November) and larger outbreak of IFV A, where IFV A(H1N1)pdm09 accounted for 33 of 34 IFV A detections. The following season, in 2010/2011, IFV A was only detected in two children, while IFV B had a higher detection rate than usual with 24 detections occurring in the common influenza season from December to April (Fig. 2).

IFV B appeared from 2006 to 2019 with varying outbreaks in a non-homogeneous pattern. Three large outbreaks in the seasons 2010/2011, 2012/2013 and 2017/2018 accounted for 58% (62/107) of all IFV B detections.

In Norway, the first detection of SARS-CoV-2 was in late February 2020. NPIs were implemented by the 12th of March 2020. Concurrently, no IFV was detected from April 2020 to January 2022, despite a doubling in nasopharyngeal tests in 2021–2022. After relieving all NPIs in February 2022, a large IFV A outbreak with 37 detections occurred in March to May 2022, comparable in size to the 2009 H1N1 influenza pandemic outbreak, though later in the season (Fig. 2). In 2022/2023 there was an even larger outbreak of IFV A with 62 detections, in addition to the largest IFV B outbreak recorded during the entire study period with 25 detections.

### Clinical and microbiological conditions before and during the 2009 H1N1 influenza pandemic

IFV positive children during the 2009 H1N1 influenza pandemic were older (median age: 49 vs. 23 months, Table 2) and more likely to have a low Ct-value than IFV positive samples collected before the pandemic (Table 2). Otherwise, there were few differences in background, clinical and microbiological variables between IFV positive children in 2006–2009 and IFV positive children in 2009/2010 (Table 2).

**Table 2:** Comparison of clinical and microbiological data of IFV children before (2006–2009) and during the 2009 H1N1 influenza pandemic in the 2009/2010 season

	2006–2009		2009/2010		Unadjusted		Adjusted	
	(*n* = 55)		(*n* = 38)					
	*n*	%	*n*	%	OR	95% CI	aOR	95% CI
**Background characteristics**								
Median age months (IQR)	23	(12–39)	49	(21–100)	**			
Age < 2 years old	28	51	11	29	**0.39**	**0.16–0.95**	**0.34** ^1^	**0.14–0.85**
Female	18	33	20	53	2.3	0.98–5.3		
Chronic disease^2^	15	27	12	32	1.2	0.50-3.0		
Premature birth^3^	13/46	28	6/33	18	0.56	0.19–1.7		
**Clinical data**								
RTI	51	93	38	100	-	-		
Sole URTI	18	35	18	47	1.7	0.70–3.9		
LRTI	33	65	20	53	0.60	0.26–1.5		
Hospitalized > 24 h	27	49	21	55	1.3	0.56–2.9		
Severe disease^4^	8	15	6	16	1.1	0.35–3.5		
LOS ≥ 5 days	7	13	5	13	1.0	0.30–3.6		
Intensive care unit	7	13	2/36	6	0.40	0.08–2.1		
Fever (temperature > 38.5 °C)	28/50	56	20	53	0.87	0.37-2.0		
Antibiotics	16	29	11	29	0.99	0.40–2.5		
Complications	16	29	10/36	28	0.94	0.37–2.4		
Severe complications	8	15	1/36	3	0.17	0.02–1.4		
Invasive group A streptococcal disease	0	0	0	0	-	-		
**Microbiological data**								
IFV A	46	84	34	89	1.7	0.47–5.8		
IFV B	9	16	4	11	0.60	0.17–2.1		
IFV Ct value < 28^5^	23/54	43	31	82	**6.0**	**2.2–15.9**	**5.9** ^**6**^	**2.1–16.3**
Single IFV detection	37	67	22	58	0.67	0.28–1.6		
Co-detection with other viruses	18	33	16	42	1.5	0.64–3.5		
Bacterial co-detection^7^	28/41	68	21/25	84	2.4	0.7–8.6		

Abbreviations: IFV, influenza virus; OR, odds ratio; CI, confidence interval; IQR, interquartile range; LRTI, lower respiratory tract infection; URTI, upper respiratory tract infection; LOS, length of stay; Ct, cycle threshold

** *p *= 0.006 by Mann-Whitney *U* test

OR and 95% CI in bold signifies statistical significance (*p*<.05 by Pearson’s Chi-Square)

^1^Adjusted detection of influenza virus A or B

^2^Chronic disease includes asthma or other lung disease, cerebral palsy, epilepsy or heart condition

^3^Gestational age < 36 weeks

^4^As defined by a severity score including hospitalization length ≥ 5 days, need for respiratory support and treatment with supplemental fluids. A “severe disease” was defined as a score ≥ 75-percentile among all virus-positive children

^5^Ct values were mainly obtained from in-house real-time PCR analysis and QIAstat-DX Respiratory panels

^6^Adjusted for age in months

^7^Hemophilus influenzae, Moraxella catarrhalis or Streptococcus pneumoniae

### Clinical and microbiological conditions before and after the COVID-19 pandemic

IFV A/B positive children referred after COVID-19 were older than children referred before COVID-19 in years 2010–2020 (median age: 62 vs. 33 months). The age difference was true for both IFV A (median 50 vs. 27 months, *p*<.001) and IFV B (median 103 vs. 70 months *p* = 0.017). Single IFV detections were more common after COVID-19 (Table 3), but there was no difference in IFV A and B Ct values.

**Table 3:** Comparison of clinical and microbiological data of IFV positive children in 2010–2020 and post COVID-19 in 2021–2023

	2010–2020		2021–2023		Unadjusted		Adjusted*	
	(*n* = 284)		(*n* = 125)					
	*n*	%	*n*	%	OR	95% CI	aOR	95% CI
**Background characteristics**								
Median age in months (IQR)	33	(14–68)	62	(22–112)	**			
Age < 2 years old	112	39	33	26	**0.55**	**0.35–0.88**	**0.48**	**0.30–0.77**
Female	124	44	51	41	0.89	0.58–1.4		
Chronic disease^1^	58	20	17	14	0.61	0.34–1.1	**0.50**	**0.27–0.91**
Premature birth^2^	32/234	14	5/51	10	0.69	0.25–1.9		
**Clinical data**								
Respiratory tract infection	280	99	120	96	0.34	0.090–1.3		
Sole upper respiratory tract infection	122	44	88	73	**3.6**	**2.2–5.7**	**3.1**	**1.9-5.0**
Lower respiratory tract infection	158	56	32	27	**0.28**	**0.18–0.45**	**0.32**	**0.20–0.53**
Hospitalized > 24 h	163	57	40	32	**0.35**	**0.22–0.54**	**0.37**	**0.24–0.59**
Severe disease^3^	50	18	11	9	**0.45**	**0.23–0.90**	0.50	0.25-1.0
Length of hospital stay ≥ 5 days	40	14	14	11	0.77	0.40–1.5		
Admitted to intensive care unit	26/282	9	4	3	**0.33**	**0.11–0.95**	**0.33**	**0.11–0.96**
Fever (temperature > 38.5 °C)	127/280	45	37	30	**0.51**	**0.32–0.79**	**0.57**	**0.36–0.90**
Antibiotics	86	30	20	16	**0.44**	**0.26–0.75**	**0.43**	**0.25–0.75**
Complications	80/282	28	37	30	1.1	0.67–1.7		
Severe complications	26/282	9	3	2	**0.24**	**0.07–0.82**	**0.24**	**0.07–0.82**
Invasive group A streptococcal disease	0	0	2	2	**-**	**-**		
**Microbiological data**								
IFV A^4^	190	67	99	79	**1.9**	**1.1–3.1**	**2.5**	**1.5–4.3**
IFV B^4^	93	33	26	21	**0.54**	**0.33–0.88**	**0.40**	**0.23–0.66**
IFV Ct value < 28^5^	220/262	84	98/124	79	0.72	0.42–1.2	0.89	0.50–1.6
Single IFV detection	210	74	106	85	**2.0**	**1.1–3.4**	1.6	0.9–2.8
Co-detection with other viruses	74	26	19	15	**0.51**	**0.29–0.7**	0.63	0.35–1.1
Bacterial co-detection^6^	114/146	78	-	-	**-**	**-**		

Abbreviations: IFV, influenza virus; OR, odds ratio; CI, confidence interval; aOR, adjusted odds ratio; IQR, interquartile range; LRTI, lower respiratory tract infection; URTI, upper respiratory tract infection; LOS, length of stay; Ct, cycle threshold

*All analyses were adjusted for age except “Age < 2 years old” which was adjusted for IFV A or B

** *p *< 0.001 by Mann-Whitney *U* test

OR and 95% CI in bold significant (*p*<.05 by Pearson’s Chi-Square)

^1^Chronic disease includes asthma or other lung disease, cerebral palsy, epilepsy or heart condition

^2^Gestational age < 36 weeks

^3^As defined by a severity score including hospitalization length > 5 days, need for respiratory support and treatment with supplemental fluids. A “severe disease” was defined as a score ≥ 75-percentile among all virus-positive children

^4^One child with both IFV A and B is not included in the IFV A and B calculations

^5^Ct values were mainly obtained from in-house real-time PCR analysis and QIAstat-DX Respiratory panels

^6^Hemophilus influenzae, Moraxella catarrhalis or Streptococcus pneumoniae

Age-adjusted logistic regression analyses revealed that fewer children referred for evaluation after the COVID-19 pandemic had chronic diseases, they less often presented with fever, were less likely to have LRTI, and less likely to be prescribed antibiotics. Complications and length of hospital stay ≥ 5 or < 5 days were comparable before and after COVID-19. However, children referred after COVID-19 were less likely to develop severe complications and fewer had severe disease (marginally non-significant) (Table 3). Sensitivity analyses revealed similar findings when we analyzed IFV A and B separately (data not shown).

### Hospitalization rates

The hospitalizations rates varied during the long study period and were highest in children < 1 years and 1–2 years of age (Supplementary Table 2).

**Fig. 3:**
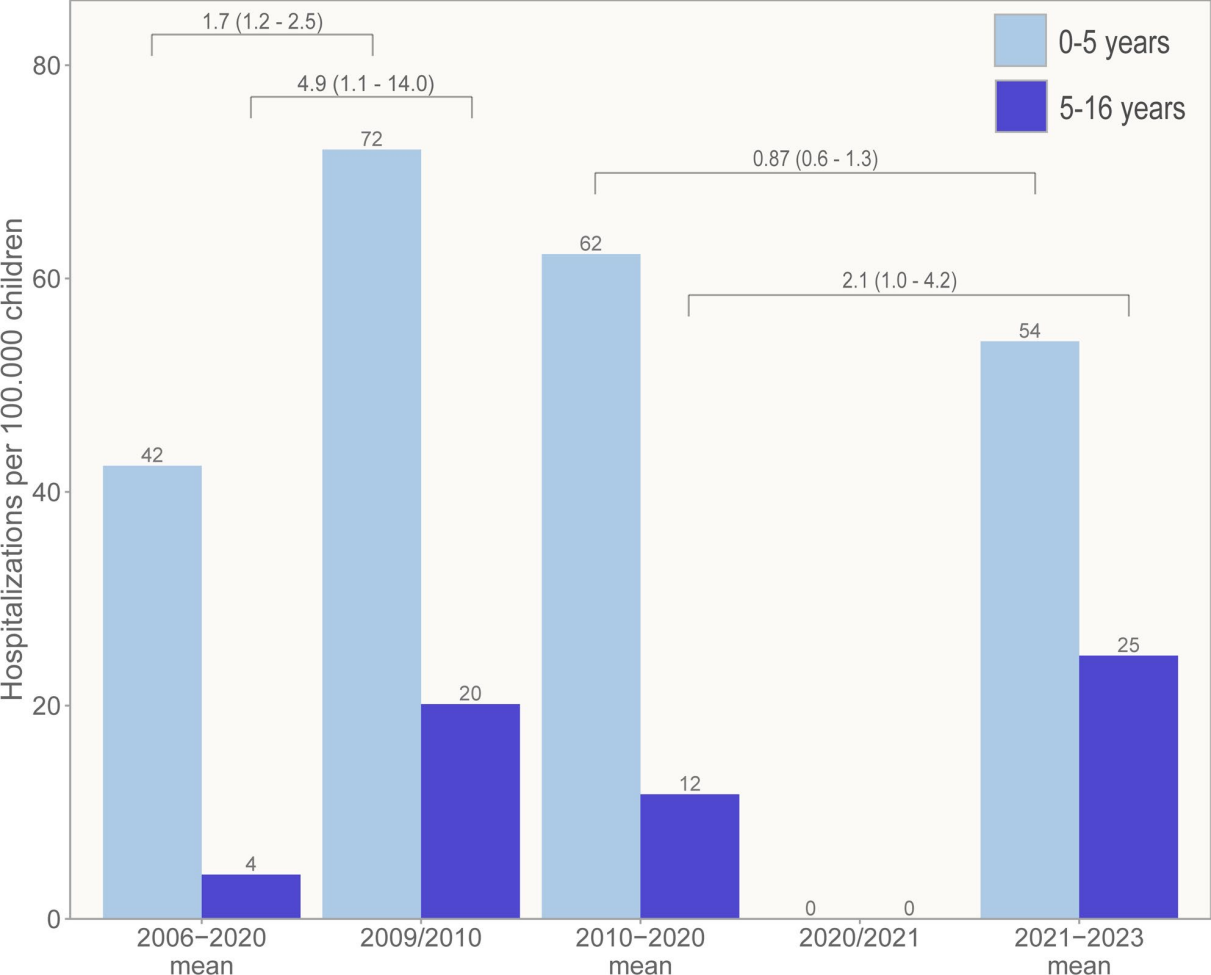
Mean hospitalization rates of influenza virus in children younger (light blue) and older (dark blue) than 5 years old. Numbers over brackets correspond to the odds ratio (95% confidence intervals) of being hospitalized with influenza versus not being hospitalized

During the 2009 H1N1 influenza pandemic the mean hospitalization rates in children ≤ 5 years and > 5 years of age increased compared to 2006–2009 (Fig. 3, Supplementary Table 2).

During COVID-19 in 2020/2021 the hospitalization rate for IFV was zero. In children ≤ 5 years it was not higher after (2021–2023) than before (2010–2020) the pandemic, but in children > 5 years it increased from pre-pandemic levels (Fig. 3, Supplementary Table 2).

## Discussion

### Main findings

During both the 2009 H1N1 influenza pandemic and the post COVID-19 years 2021–2023 there was an increase in number of IFV A/B positive nasopharyngeal samples collected from children referred for evaluation at the hospital compared to regular influenza seasons. Hospitalization rates increased for all age categories during the 2009 H1N1 influenza pandemic. Post-COVID-19, however, the rate only increased in children > 5 years old. For both periods, children were on average older, with a doubled age compared to regular influenza seasons. Disease severity during the 2009 H1N1 influenza pandemic was comparable to earlier years, whereas children post-COVID-19 developed less severe disease compared to the years prior to COVID-19. Our data support that novel IFV A(H1N1)pdm09 in 2009/10 and seasonal IFV A and B in 2022–2023 caused more infections in need of hospital referral because of a lack of immunity, but the viruses were not likely to be more pathogenic or cause more severe infections than previous seasonal IFV strains.

### Influenza seasonality from year 2006 to 2023

Similarly to previous studies conducted in the Northern Hemisphere, we observed during the 17 year-long study period from 2006 to 2023 that seasonal IFV occurred in yearly winter epidemics from November to March [18], with two exceptions. During early fall in 2009 we detected a large outbreak of pandemic IFV A(H1N1)pdm09, which also appeared in many other places in the world, explained by insufficient immunity in young individuals. Surprisingly, in the next season 2010/2011 IFV A(H1N1)pdm09 disappeared, but instead a large IFV B outbreak occurred. This finding contradicts studies from other countries where IFV A (including (H1N1)pdm09) also dominated in the 2010/2011 season [19–21]. In Norway, there was a high vaccination coverage with *Pandemrix* in 2009/10 with nearly half of the population vaccinated across all age groups [22]. Analyses of population sera have revealed unusually high percentages (22–26%) of seropositive samples against both IFV A(H1N1)pdm09 and IFV A(H3N2) in August 2010 [23]. At the same time, the seropositivity against IFV B (Brisbane) was low at 10% [23]. Thus, it has been speculated that there was a strong immunity against especially IFV A(H1N1)pdm09 and IFV A(H3N2) in the fall of 2010, and then instead IFV B became the dominating virus in the season 2010/2011 [23]. After 2010/2011, IFV A(H1/N1)pdm09 strains have circulated as seasonal viruses in conjunction with various IFV A(H3N2) and IFV B strains.

Concurrent with the arrival of SARS-CoV-2 in Norway and the implementation of NPIs, we detected no IFV during a 21-month long period from April 2020 to January 2022, similar to other countries [8, 9]. In the two following seasons (2021/2022 and 2022/2023) we observed three IFV outbreaks at atypical time points. First, a late IFV A outbreak in March to May 2022, and then in 2022/2023 an early and larger IFV A outbreak in October to December 2022, followed by a large IFV B outbreak in January to May 2023. While virus interactions between IFV and SARS-CoV-2 may play a role [24], the primary driver behind reduction in virus transmission of all respiratory viruses in 2020/2021 is likely to be changes in social relations and behavior [24, 25]. Recently, it was shown in a Norwegian study that the youngest children had lower protecting antibody levels against the dominating IFV A(H3/N2) strain in 2021 and the dominating A(H1N1)pdm09 strain in 2022 [15]. This “immunity gap” is likely to be the result of absent exposure to these viruses in 2020/2021, and might be one factor explaining that more children were referred for evaluation at our hospital after COVID-19 in 2022/2023, and that more children > 5 years of age were treated as in-patients [26–30].

### Influenza severity during the 2009 H1N1 influenza pandemic and post COVID-19 influenza outbreaks

There were minimal distinctions in clinical manifestations and short-term outcomes when we compared pre-pandemic children with IFV A referred in 2006–2009 to children with the novel IFV A(H1N1)pdm09 in 2009/2010, except that children infected with A(H1N1)pdm09 were on average double as old. We speculate that higher average age might be attributable to a lack of immunity in children at all ages leading to more novel A(H1N1)pdm09 infections. Previous studies have reported that IFV A(H1N1)pdm09 in 2009/2010 caused milder infections than influenza A(H3N2) in the general population [31], whereas a review article from 2014 summarizes a large and diverse literature and suggests that hospitalized children on average had worse outcomes than before, although large variations related to age, premorbid conditions, clinical presentations and complications were found [32].

Much like the 2009 H1N1 influenza pandemic, we found that referred children with seasonal IFV after the COVID-19 pandemic in 2021–2023 on average were older compared to regular influenza seasons. This observation is consistent with findings from studies across the globe [13, 33, 34], and is generally regarded as “filling of the immunity gap” that resulted from the absence of circulation of common respiratory viruses during COVID-19 due to NPIs. However, more children were previously healthy, and our data clearly indicate that they on average developed less severe influenza, e.g. fewer children had fever, LRTI, antibiotic treatment, high clinical severity score, intensive care treatment and severe complications. These observations support that seasonal IFV after COVID-19 at least did not cause more severe influenza despite more children having increased susceptibility.

There is already much documentation of the resurgence of respiratory viruses after all NPIs were lifted post-COVID-19. E.g. after absent RSV in 2020/2021, RSV outbreaks in the early fall in 2021 caused LRTI of mainly unchanged severity whereas large RSV outbreaks in 2022/2023 were reported to cause more severe LRTI in infants but also in 1–4 years old children [35, 36]. When it comes to influenza, in contrast to our findings, a few recent reports suggest increased severity of seasonal IFV compared to pre-COVID-19 years. At the society level in US in the 2022/2023 season, number of influenza-like infections and hospitalizations, but also mortality increased in children < 14 years [13]. A prospective pediatric influenza cohort in Nicaragua reported that in the season 2022/2023 IFV A(H3N2) was associated with older age, higher incidence among 5–14 years old children and more children aged 0–4 years with severe disease compared to the pre-pandemic seasons 2017/2018 and 2019/2020 [16]. A multicenter retrospective study from tertiary health care in China showed that an 2023/2024 IFV A(H1N1) outbreak caused more severe infections compared to pre-pandemic years 2018–2019 as evidenced by longer and higher fevers, more cough, more seizures, longer hospital stays and increased numbers with WHO-defined COVID-19 severe infections [37]. However, another multicenter study from China comparing pediatric influenza hospitalizations in the years 2018–2019 with 2020–2023 found shorter hospitalizations and lesser hospitalization expenses in the post-pandemic years [38], but there was a tendency towards greater use of oxygen and respiratory support without a corresponding increase in the duration of supplementary oxygen or respiratory support [39]. IFV causes a variety of manifestations in children at various ages, and more information from other countries and settings are needed before it is possible to make firm conclusions about seasonal IFV severity in the post-COVID-19 period.

An “immunity gap” due to less exposure of IFV during the NPIs in 2020 and 2021 might explain that in this study, we detected more children referred to hospital with older age and milder IFV disease post-COVID-19 [15, 26–30]. However, we have considered other explanations for these findings. First, we cannot exclude the possibility that changes in parents and children’s behaviors after COVID-19 due to worries and fear of complications could explain admittance of more children with milder disease. Unfortunately, we have no data available to shed light on the handling in primary health care before admittance to hospital. Secondly, there was a more liberal sampling for respiratory viruses post-COVID-19 compared to pre-pandemic years, which might have resulted in an increase of IFV detections in less sick children. We have not adjusted for sampling frequency; however, we noticed that the sampling frequency increased prior to the increased detection rate of IFV as illustrated in Fig. 3. Thirdly, specimen collection of nasopharyngeal secretes changed over time from aspirates to swaps, and PCR-tests changed from in-house to commercial multiplex respiratory virus panels. The effects of these changes on age and disease severity distributions are uncertain, but most likely limited. It is worth noting that prior to and during the 2009 H1N1 influenza pandemic, only nasopharyngeal aspirates and in-house PCR tests were used, and still the age of IFV positive children doubled, while disease severity was unchanged. Fourth, fluctuations in IFV vaccination coverage over time influence the number infected children and possibly age distribution and disease severity. In Norway IFV vaccination during the entire study period has only been recommended for children with risk factors such as chronic diseases. Hence, the vaccination rate in children without premorbid conditions is negligible and it has been reported to be less than one of ten with risk factors [15]. Finally, it is well-known that younger age is related to severe diseases in children with influenza, but in this study our findings were adjusted for age in the analysis. Nevertheless, during the entire study period, several children at a young age and children with chronic diseases developed severe influenza in need of hospital admission and intensive care. The low IFV vaccination rate in Norwegian children with risk factors is worrying and efforts should be raised to increase the rate, but the substantial impact of IFV among healthy children should also raise considerations to include IFV vaccination in the National Immunization Program for Children. At least our findings point toward the need to consider a general IFV vaccination strategy for all children in future pandemics with NPIs.

### Strengths and limitations

Although this is a single center observational study, it is a strength that the results are based on a longitudinal dataset of children with confirmed IFV enrolled over 17 consecutive years at a hospital serving as the sole pediatric center for the entire pediatric population in Sør-Trøndelag county, Norway. It allows particularly comparisons of post-COVID-19 data with 2010–2020 data, avoiding errors due to random seasonal differences. In the absence of a validated severity score to assess acute RTI in children we have used a self-composed severity score in this and other published studies. This score reflects clinical routines at our pediatric department [35]. As influenza may lead to other complications than those related to RTIs we included other severity indicators such as the rate with LRTI, length of hospital stays and prescription of antibiotics. The study was performed in a hospital setting and is not representative for a pediatric population outside the hospital. Our results are probably generalizable to other hospitals in the Northern Hemisphere, and the long study period compensates for seasonal variances. It is a limitation that we have no information about IFV strains, vaccination rates and antiviral treatment. Premature birth was defined in the study form as gestational age < 36 weeks, rather than < 37 weeks.

## Conclusion

Compared to regular influenza seasons, more children were referred to a secondary evaluation at the hospital, and more were hospitalized > 24 h during the 2009 pandemic. Post-COVID-19 more children were referred for evaluation and children > 5 years had increased hospitalization rate. During the 2009 H1N1 influenza pandemic disease severity was unchanged, but post-COVID-19 children on average had a milder disease.

## Supplementary Information

Below is the link to the electronic supplementary material.

Supplementary Material 1

## Data Availability

The datasets generated and analyzed during the current study are not publicly available due to individual privacy.

## Abbreviations

IFV: Influenza virus
LRTI: Lower respiratory tract infection
URTI: Upper respiratory tract infection
NPIs: Non-pharmacological interventions
PICU: Pediatric intensive care unit
