# Supplementary material for: Hospitalized children with influenza virus: a 17 year-long observational study including the 2009 H1N1 influenza pandemic and COVID-19 pandemic
Source: BMC Infect Dis. 2026 Feb 12;26:577. doi: 10.1186/s12879-026-12818-5 (PMC12998135; doi:10.1186/s12879-026-12818-5)

**Supplementary materials**

| **Supplementary table 1.** Complications among hospitalized children and outpatiens with influenza before and after COVID-19. | | | | |
| --- | --- | --- | --- | --- |
|  | 2010-2020 |  | 2021-2023 |  |
|  | n | % | n | % |
| **Hospitalized children admitted to ICU** | **26/163** | **16** | **4/40** | **10** |
| Previously healthy | 15/26 | 58 | 3/4 | 75 |
| Underlying chronic condition | 11/26 | 42 | 1/4 | 25 |
| **Hospitalized > 24 hours** |  |  |  |  |
| **Mild complications** | **21/163** | **13** | **10/40** | **25** |
| Febrile seizure | 10/21 | 48 | 3/10 | 30 |
| Mild CNS symptoms | 2/21 | 10 | 1/10 | 10 |
| Facial paralysis | 0 | - | 0 | - |
| Skin rash | 1/21 | 5 | 1/10 | 10 |
| Myositis | 2/21 | 10 | 1/10 | 10 |
| Dehydration | 2/21 | 10 | 0 | - |
| Other mild complications | 4/21^1^ | 19 | 4/10^2^ | 40 |
| **Severe complications** | **26/163** | **16** | **3/40** | **8** |
| Sepsis like disease | 2/26 | 8 | 0 | - |
| Severe complicated RTI | 17/26^3^ | 65 | 1/3^4^ | 33 |
| Severe CNS complications | 4^5^ | 15 | 0 | - |
| Severe soft tissue complications | 0 | - | 0 | - |
| Acute arthitis and osteomyelitis | 0 | - | 0 | - |
| Other severe complications | 3^6^ | 12 | 2/3^7^ | 67 |
| **Outpatients** |  |  |  |  |
| **Mild complications** | **33/121** | **27** | **24/85** | **28** |
| Febrile seizure | 10/33 | 30 | 6/24 | 25 |
| Mild CNS symptoms | 5/33 | 15 | 5/24 | 21 |
| Facial paralysis | 1/33 | 3 | 1/24 | 4 |
| Skin rash | 7/33 | 21 | 3/24 | 13 |
| Myositis | 6/33 | 18 | 5/24 | 21 |
| Dehydration | 2/33 | 6 | 4/24 | 17 |
| Other mild complications | 2/33^8^ | 6 | 0 | - |

Abbreviations: CNS, central nervous system; ICU, intensive care unit; RTI, respiratory tract infection.

^1^Including two children with neutropenia and two with preseptal cellulitis. ^2^One with IgA nephritis, one with anemia, one with osteomyelitis (*Streptococcus aureus*), one influenza and SARS-CoV-2 positive child with MIS-C. ^3^Including one child with ARDS, one child with a rare syndrome in need of intensive care diagnosed with laryngitis, one total atelectasis, one bacterial tracheitis (*Streptococcus pneumoniae*), one with secondary bacterial pneumonia and pneumatocele (unknown bacteria). ^4^*Streptococcus pyogenes* pneumonia. ^5^Including four children with complicated seizures. ^6^Including one child with severe glomerulonephritis, one with periorbital cellulitis and one with hematemesis. ^7^One child with Lemierres syndrome (*Streptococcus pyogenes*) and one with preorbital cellulitis. ^8^One with leukopenia and one with ethmoidal sinusitis.

**Supplementary table 2.** Children with influenza virus hospitalized >24 hours per 100.000 children in Sør-Trøndelag county.

| Epidemiological year |  | <12 months | 12-23 months | 24-59 months |  | <60 months | >60 months |  | All ages |
| --- | --- | --- | --- | --- | --- | --- | --- | --- | --- |
| 2006/2007 |  | 87 | 86 | 59 |  | 70 | 2 |  | 23 |
| 2007/2008 |  | 56 | 58 | 10 |  | 29 | 5 |  | 12 |
| 2008/2009 |  | 28 | 111 | 0 |  | 28 | 5 |  | 12 |
| 2009/2010 |  | 106 | 111 | 47 |  | 72 | 20 |  | 36 |
| 2010/2011 |  | 79 | 0 | 18 |  | 27 | 15 |  | 19 |
| 2011/2012 |  | 26 | 53 | 36 |  | 38 | 8 |  | 17 |
| 2012/2013 |  | 300 | 156 | 27 |  | 107 | 15 |  | 45 |
| 2013/2014 |  | 137 | 108 | 26 |  | 64 | 8 |  | 26 |
| 2014/2015 |  | 82 | 55 | 9 |  | 32 | 8 |  | 15 |
| 2015/2016 |  | 187 | 189 | 36 |  | 96 | 0 |  | 31 |
| 2016/2017 |  | 54 | 53 | 63 |  | 59 | 30 |  | 39 |
| 2017/2018 |  | 134 | 189 | 36 |  | 86 | 15 |  | 37 |
| 2018/2019 |  | 83 | 27 | 79 |  | 70 | 10 |  | 28 |
| 2019/2020 |  | 30 | 138 | 18 |  | 44 | 10 |  | 20 |
| 2020/2021 |  | 0 | 0 | 0 |  | 0 | 0 |  | 0 |
| 2021/2022 |  | 30 | 86 | 28 |  | 40 | 9 |  | 18 |
| 2022/2023 |  | 27 | 149 | 57 |  | 69 | 40 |  | 48 |
| Mean |  | 85 | 92 | 32 |  | 55 | 12 |  | 25 |
| 95% confidence interval |  | 50-119 | 65-120 | 22-43 |  | 42-68 | 7-17 |  | 19-31 |
|  | | | | | | | | | |

**Supplementary figure 1.** Study flowchart in line with the STROBE (Strengthening the Reporting of Observational Studies in Epidemiology) statement.


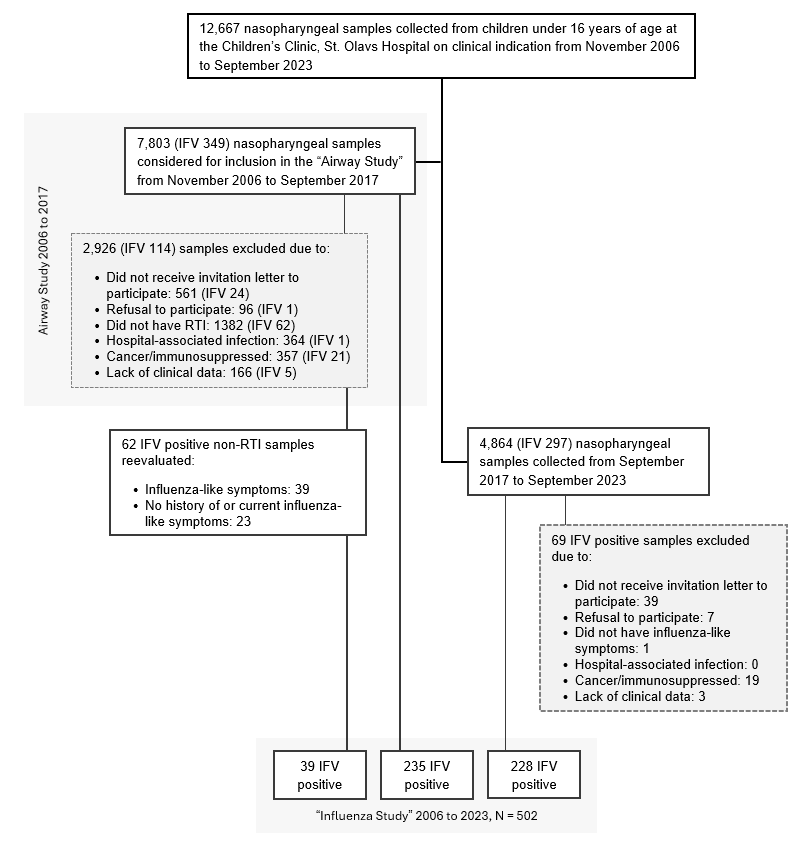

Supplement: Supplementary file 1 — Supplementary Material 1 [file 12879_2026_12818_MOESM1_ESM.docx]
